# Supplementary material for: PD-1 Expression Promotes Immune Evasion in B-ALL
Source: Hematol Rep. 2025 Nov 12;17(6):61. doi: 10.3390/hematolrep17060061 (PMC12641702; doi:10.3390/hematolrep17060061)
Supplement: Supplementary file 1 [file hematolrep-17-00061-s001.zip › hematolrep-3738887-supplementary.pdf]

Supplemental information for:

PD-1 Expression Promotes Immune Evasion in B-ALL

INDEX:

1. **Methods**
2. **Supplemental Tables**
3. **Supplemental Figures**

1- Methods

Antibiotic treatment

Mice were treated with an antibiotic regimen as described elsewhere [1]. Briefly, cohorts of mice were given a cocktail of antibiotics (ampicillin, 1 g/L, Ratiopharm; vancomycin, 500 mg/L, Cell Pharm; ciprofloxacin, 200 mg/L, Bayer Vital; imipenem, 250 mg/L, MSD; metronidazole, 1 g/L, Fresenius), which was added to their drinking water *ad libitum* for a period of eight weeks.

Bone marrow aspirated samples

Bone marrow samples were obtained from the femurs of anesthetized mice at 3, 6, 9 and 12 months. We used Sevorane (Abbvie, CAS #28523-86-6 _ Sigma_ Merck Life Science S.L.U. Madrid 28006, SPAIN) as anaesthetic and sterile 27G syringes for the bone marrow aspirates (BD Plastipak; ref 305503_BD 28750, San Agustin de Guadalix, Madrid, SPAIN). Samples were obtained in fresh 2% FCS PBS. Then, samples were washed with PBS (5′, 3000 g, RT) and the supernatant discarded. Cell pellets were resuspended in 1 ml of red cell lysis buffer to eliminate contaminating red blood cells. After 15 minutes, samples were transferred to a 50ml falcon tube containing 5 ml of PBS through a 70 µm cell strainer, then washed with 4 ml of PBS (centrifuged for 5′ at 1400 rpm, at 4ºC). Supernatants were discarded and cell pellets were resuspended in 400 µl of PBS/pellet. At that point, 100 µl of each sample were used for flow cytometric analysis.

Spectral cytometry FACS analysis

Spectral flow cytometric analysis of murine B-ALL

Nucleated cells were obtained from total mouse bone marrow, peripheral blood, thymus, lymph nodes and/or spleen. Contaminating red blood cells were lysed with red cell lysis buffer (RCLB) and the remaining cells were washed in PBS. Samples were stained with the fixable Zombie NIR viability dye kit (BioLegend; 423105_ Palex Medical S.A. Barcelona 08174-Spain) following the manufacturer's instructions. Subsequently, cells were washed with PBS supplemented with 1% FCS to eliminate the remaining viability marker (5′, 1400 rpm, RT). Then, samples were incubated with anti-CD16/32 (BD Biosciences, 553142_BD 28750, San Agustin de Guadalix, Madrid, SPAIN) at a concentration of 1:100 (5 minutes at RT) to suppress nonspecific antibody binding. After washed, the samples were stained with the mix of antibodies detailed below for 20 minutes at 4ºC in the darkness. Prior to acquisition in the flow cytometer, samples were washed with 1% FCS PBS to eliminate the excess of antibody. Samples were acquired in a Cytek Northern Lights 2000 spectral cytometer (two lasers, red and blue) and analyzed with FCS Express software. Specific fluorescence of the fluorophores, as well as known forward and orthogonal light scattering properties of mouse cells, were used to establish gates. For each analysis, a total of 100,000 cells were assessed. The following antibodies and concentrations were used for flow cytometry: anti-CD8a (BD Biosciences 564459; 1/100), anti-CD4 (eBioscience 58-0042-82; 1/100), anti-PD1 (Biolegend 135206; 2/100), anti-CD25 (BioLegend 102048; 2/100), anti- CD11b (BioLegend 101280; 1/100), anti-NK1.1 (anti-CD161) (BioLegend 108715; 1;177), anti-CD19 (BioLegend115534; 4/100), andi-CD49b (eBiosceince 46-5971-82; 1/100), anti-PDL1 (BioLegend 155406; 1/100), anti-B220 (BioLegend 103287; 1/100), anti-IgM (BioLegend 406509; 1/100), anti-Ly6G/Ly6C (BioLegend 108418; 0.5/100), anti-CD117 (BD Bioscience 565476; 1100), anti-CD45 (BioLegend 147713; 1/100), anti-Ter119 (BioLegend 116264; 1/100).

Samples from WT mice reconstituted with leukemic *Pax5*$^{+/-}$ cells were analyzed differently. After red blood cell lysis, remaining cells were washed in PBS with 1% FCS. Then, samples were incubated with anti-CD16/32 (BD Biosciences, 553142) at a concentration of 1:100 for 5 minutes at RT to supress the nonspecific antibody binding. Later, samples were stained with the mix of antibodies detailed below for 20 minutes at 4ºC in the dark. Prior to acquisition in the flow cytometer, samples were washed with 1% FCS PBS to eliminate the excess of antibody and then resuspended in PBS with 1% FCS containing 10 μg/mL propidium iodide (PI) to exclude dead cells from further analysis. The following antibodies and concentrations were used for flow cytometry: anti-B220 (RA3-6B2, 1/100, cat. 103212), CD4 (RM4-5, 1:250, cat. 100516), CD8a (53-6.7, 1:250, cat. 100708), CD11b/Mac1 (M1/70, 1:200, cat. 553310), CD19 (1D3, 1/100, cat. 152404), CD117/c-Kit (2B8, 1:200, cat. 105807), Gr1 (RB6-8C5, 1/100, cat. 108412), IgM (R6-60.2, 1/100, cat. 406509) and CD25 (PC61, 1/100, cat. 553866) antibodies. All antibodies were purchased from BioLegend-_ Palex Medical S.A. Barcelona 08174-Spain or BD Biosciences, BD 28750, San Agustin de Guadalix, Madrid, SPAIN, as indicated.

Spectral flow cytometric analysis of human B-ALL

A total of 57 childhood B-ALL samples collected at diagnosis under an IRB approved protocol and informed consent, were freshly frozen in multiple aliquots. One aliquot per patient was subsequently thawed at 37ºC. Thawed samples were washed twice in 2 mL of phosphate buffered saline (PBS, pH=7.4) and immediately stained with the Fixable Viability Stain reagent FVS780 -Becton/Dickinson Biosciences (BD), San Jose, CA- following the manufacturer's instructions. Cells were washed once with PBS and stained with one of the two monoclonal antibody combinations (panel A or panel B) detailed in Supplemental Table 3, using the EuroFlow sample preparation and staining for cell surface markers-only, standard operating procedures (SOP) available at www.EuroFlow.org. Stained cells were placed in 500 L of PBS and immediately acquired in an Aurora spectral flow cytometer (Cytek Northern Light 3000, Cytek Biosciences, Fremont, CA). For data analysis the Infinicyt™ software v2.0.5.e.000 (Cytognos SL, Salamanca, Spain) was used. PD-1 and PD-L1-associated fluorescence was reported specifically for $CD19^{+}$ vs $CD45^{low}$ gated blast cells, after subtracting the cellular background fluorescence, as described elsewhere [2].

Histology

Tissue samples were fixed in formalin and embedded in paraffin. Pathology assessment was performed on hematoxylin-eosin stained sections under the supervision of Dr. Oscar Blanco, an expert pathologist at the University Hospital of Salamanca (Spain).

Whole-genome sequencing

Tumor DNA was derived from the BM where the percentage of blast cells was higher than 80%. Germline DNA was obtained from the tail when the mice were 4-weeks old (pre-leukemic). Genomic DNA libraries were prepared from sheared DNA with the HyperPrep Library Preparation Kit (Roche PN 07962363001_Roche Diagnostics_Barcelona_Spain). Paired-end 150 cycle sequencing was performed on a NovaSeq 6000 (Illumina). Illumina paired-end reads were preprocessed and were mapped to the mouse reference genome (mm10) with BWA [3]. We used an ensemble approach to call somatic mutations (SNV/indels) with multiple published tools, including Mutect2 [4], SomaticSniper [5], VarScan2 [6], MuSE [7], and Strelka2 [8]. The consensus

calls by at least two callers were considered as confident mutations. The consensus call sets were further manually reviewed for read depth, mapping quality, and strand bias to remove additional artifacts. Somatic copy-number alternations were determined by CNVkit [9]. For somatic structural variants (SV), four SV callers were implemented in the workflow for SV calling, including Delly [10], Lumpy [11], Manta [12], and Gridss [13]. The SV calls passing the default quality filters of each caller were merged using SURVIVOR [14], and genotyped by SVtyper (v0.1.4) [15]. The intersected call sets were manually reviewed for the supporting soft-clipped and discordant read counts at both ends of a putative SV site using IGV.

Whole-exome sequencing

Whole exome sequencing (WES) was performed using the Illumina TruSeq Exome Enrichment Kit (Thermo Fisher Scientific Inc. Alcobendas (Madrid), SPAIN). For WES, samples are sequenced to obtain approximately 65% of bases at 45X depth of coverage. Illumina paired-end reads were preprocessed and were mapped to the mouse reference genome (mm10). We used above-described ensemble approach to call somatic single nucleotide variants (SNV/indels) with multiple published tools, including Mutect2, SomaticSniper, VarScan2, MuSE and Strelka2. The consensus calls by at least two callers were considered as confident mutations. Mutations were further filtered using quality filters for the read depth, mapping quality, and strand bias to remove low quality calls.

NK cytotoxicity assay

Ex vivo murine NK cell cytotoxicity assay

NK cell cytotoxicity assay on leukemic proB cells (B220$^+$ PD-1$^+$) from a disease Pax5$^{+/-}$ mouse (S748) and on preleukemic Pax5+/- proB cells (B220$^+$ CD25$^+$) was carried out as previously described [16] with minor modifications. Eight-twelve weeks old wild-type mixed C57BL/6× CBA background mice (n=6) were used in this study as the source of the NK cells. Those mice were injected intraperitoneally with 300ug poly(I:C) in PBS for each mouse 18-24h before NK purification, to activate NK cells. Next, NK cells were enriched using the mouse NK Cell Isolation kit (Miltenyi Biotec; cat #130-115-818_ Milteny Biotec S.L. 28223 Pozuelo de Alarcón (Madrid) Spain) from pooling spleens mice to ensure sufficient NK cell numbers for the killing assay. Once NK cells had been isolated, the killing assay was done by co-cultured activated NK cells with leukemic (S748) or preleukemic Pax5$^{+/-}$ proB cells at a ratio of 1:1 for 8 hours in pro-B-cell medium (IMDM medium + L-glutamine + HEPES, 2% FCS, 1 mM penicillin–streptomycin, 0.03% Primaton, 50 μM beta-mercaptoethanol, 2 μg/ml cyproxin; Thermo Fisher Scientific Inc. Alcobendas (Madrid), SPAIN) supplemented with 5 ng/ml murine recombinant IL-7 (ImmunoTools cat #12340073_ ImmunoTools, Gladiolenweg 2, 26169 Friesoythe, Germany) and 10 ng/ml of murine IL-15 (peprotech cat: #210-15; Thermo Fisher Scientific Inc. Alcobendas (Madrid), SPAIN) in a 12 well plate at a final volume of 2 ml/well. Cells were treated with or without (vehicle) anti-PD1 monoclonal antibody (BioXCell; cat #BE0146; 7.36 μg/mL; Abyntek Biopharma, S.L. 48160 Derio – Bizkaia (Spain)) or the anti-PD-1 monoclonal antibody that lacks the ability to bind to Fc receptors (Fc-silent RMP1-14; Absolute antibody; cat #Ab00813-4,3; Abyntek Biopharma, S.L. 48160 Derio – Bizkaia (Spain)) for 8 hours before being subjected to the cell viability assay, as described above. The difference between experimental groups was determined using an unpaired t-test as follows: Antibody dependent cellular cytotoxicity

(ADCC) using a PD-1 monoclonal antibody was quantitated by flow cytometry (n=3). An unpaired t-test was used to detect differences between the two conditions of culture. Error bars represent the mean and standard deviation as shown in Figure 5C. The ADCC using an Fc-silent PD-1 monoclonal antibody was quantitated by flow cytometry (n=3). An unpaired t-test was used to evaluate the differences between the two conditions as shown in Figure 5H.

Ex vivo human NK cell cytotoxicity assay

The NK cell cytotoxicity assay was carried out using the human leukemic B-ALL REH cells. REH human cell line was used from ATCC (CRL-8286_ https://www.atcc.org/products/crl-8286). Optical genome mapping was used to authenticate REH cell line as acute lymphoblastic leukemia cells. REH human cell line was tested negative for mycoplasma contamination. The *PAX5*-mutant REH cell line was derived from a primary clonal culture isolated from pre-B ALL peripheral blood [17] and contains a heterozygous p.A322fs *PAX5* null mutation [18]. The NK cell cytotoxicity assay was carried out as described [19] with minor modifications. The *PAX5*-mutant REH cells were labeled with carboxyfluorescein succinimidyl ester (CFSE) dye (Invitrogen_Thermo Fisher Scientific Inc. Alcobendas (Madrid), SPAIN) for 15min at 37ºC in the dark and washed twice with complete media to quench the labeling reaction. NK cells were isolated from a buffy coat by NK cell isolation kit (Miltenyi, Ref: 130-092-657_ Milteny Biotec S.L. 28223 Pozuelo de Alarcón (Madrid) Spain). Cells were cultured at $1.0x10^6$ cells/mL in NK MACS Basal Medium (Miltenyi, REF: 130-112-968_ Milteny Biotec S.L. 28223 Pozuelo de Alarcón (Madrid) Spain) supplemented with IL15 (10 ng/mL) (Peprotech, REF: 200-15), IL12 (10ng/ml) (R&D 219-IL) and IL18 (50ng/ml) (R&D 9124-IL) for 16 hours. CD25 expression was measured to analyse NK activation by flow cytometry. After 16 hours of activation, NK cells were washed to remove interleukins and were cultured in NK MACS medium. REH cells and K-562 cells were grown in RPMI medium (Gibco) supplemented with 10% FBS (Gibco) and 1% P/S (Gibco). Both cells were stained with a final concentration of 0.1 μM CFSE (Thermo Fisher Scientific, REF: C34554_ Thermo Fisher Scientific Inc. Alcobendas (Madrid), SPAIN) for 15 minutes at 37ºC in the dark. The cells were washed with RPMI medium and cultured in NK MACS medium. GoInVivo Purified anti-human CD279 (PD1) (Biolegend, REF: 329946) was added to the REH cells at a final concentration of 1μg/1μL and incubated for 30 minutes before the addition of NK cells. The culture was centrifuged at 240g for 4 minutes. After 8h 7AAD (5μl/sample) was added before acquisition in the BD FACSCanto II flow cytometer. The percentage of dead cells was calculated using the FlowJo software. Differences between experimental groups were determined using a Wilcoxon-test as follows: human REH cells were co-cultured with human NK cells at different effector:target ratios in the presence (red) or absence (green) of anti-PD1 for 8h (n=3). Antibody dependent cellular cytotoxicity (ADCC) was quantitated by flow cytometry. Mean and mean standard errors are shown in Figure 5E. Wilcoxon test p-value = 0.05.

Statistical analyses

Sample sizes were determined based on the literature describing mouse modeling of natural infection-driven leukemia [20-25] and were justified by power calculations estimating 90% power to detect differential leukemia incidence. Statistical analyses were performed using GraphPad Prism v8.2.1 (GraphPad Software). Statistical significance was calculated by two-tailed unpaired *t* test after data normality was tested using the D'Agostino and Pearson test (alpha level = 0.05). Survival analyses were performed using the log-rank/Mantel–Cox test.

References

1. Cording, S.; Fleissner, D.; Heimesaat, M.M.; Bereswill, S.; Loddenkemper, C.; Uematsu, S.; Akira, S.; Hamann, A.; Huehn, J. Commensal microbiota drive proliferation of conventional and Foxp3(+) regulatory CD4(+) T cells in mesenteric lymph nodes and Peyer's patches. *Eur J Microbiol Immunol (Bp)* **2013**, *3*, 1–10. https://doi.org/10.1556/EuJMI.3.2013.1.1.
2. Brodska, B.; Otevrelova, P.; Salek, C.; Fuchs, O.; Gasova, Z.; Kuzelova, K. High PD-L1 Expression Predicts for Worse Outcome of Leukemia Patients with Concomitant NPM1 and FLT3 Mutations. *Int J Mol Sci* **2019**, *20*. https://doi.org/10.3390/ijms20112823.
3. Li, H.; Durbin, R. Fast and accurate long-read alignment with Burrows-Wheeler transform. *Bioinformatics* **2010**, *26*, 589–595. https://doi.org/10.1093/bioinformatics/btp698.
4. Cibulskis, K.; Lawrence, M.S.; Carter, S.L.; Sivachenko, A.; Jaffe, D.; Sougnez, C.; Gabriel, S.; Meyerson, M.; Lander, E.S.; Getz, G. Sensitive detection of somatic point mutations in impure and heterogeneous cancer samples. *Nat Biotechnol* **2013**, *31*, 213–219. https://doi.org/10.1038/nbt.2514.
5. Larson, D.E.; Harris, C.C.; Chen, K.; Koboldt, D.C.; Abbott, T.E.; Dooling, D.J.; Ley, T.J.; Mardis, E.R.; Wilson, R.K.; Ding, L. SomaticSniper: Identification of somatic point mutations in whole genome sequencing data. *Bioinformatics* **2012**, *28*, 311–317. https://doi.org/10.1093/bioinformatics/btr665.
6. Koboldt, D.C.; Zhang, Q.; Larson, D.E.; Shen, D.; McLellan, M.D.; Lin, L.; Miller, C.A.; Mardis, E.R.; Ding, L.; Wilson, R.K. VarScan 2: Somatic mutation and copy number alteration discovery in cancer by exome sequencing. *Genome Res* **2012**, *22*, 568–576. https://doi.org/10.1101/gr.129684.111.
7. Fan, Y.; Xi, L.; Hughes, D.S.; Zhang, J.; Futreal, P.A.; Wheeler, D.A.; Wang, W. MuSE: Accounting for tumor heterogeneity using a sample-specific error model improves sensitivity and specificity in mutation calling from sequencing data. *Genome Biol* **2016**, *17*, 178. https://doi.org/10.1186/s13059-016-1029-6.
8. Kim, S.; Scheffler, K.; Halpern, A.L.; Bekritsky, M.A.; Noh, E.; Kallberg, M.; Chen, X.; Kim, Y.; Beyter, D.; Krusche, P.; et al. Strelka2: Fast and accurate calling of germline and somatic variants. *Nat Methods* **2018**, *15*, 591–594. https://doi.org/10.1038/s41592-018-0051-x.
9. Talevich, E.; Shain, A.H.; Botton, T.; Bastian, B.C. CNVkit: Genome-Wide Copy Number Detection and Visualization from Targeted DNA Sequencing. *PLoS Comput Biol* **2016**, *12*, e1004873. https://doi.org/10.1371/journal.pcbi.1004873.
10. Rausch, T.; Zichner, T.; Schlattl, A.; Stutz, A.M.; Benes, V.; Korbel, J.O. DELLY: Structural variant discovery by integrated paired-end and split-read analysis. *Bioinformatics* **2012**, *28*, i333–i339. https://doi.org/10.1093/bioinformatics/bts378.
11. Layer, R.M.; Chiang, C.; Quinlan, A.R.; Hall, I.M. LUMPY: A probabilistic framework for structural variant discovery. *Genome Biol* **2014**, *15*, R84. https://doi.org/10.1186/gb-2014-15-6-r84.
12. Chen, X.; Schulz-Trieglaff, O.; Shaw, R.; Barnes, B.; Schlesinger, F.; Kallberg, M.; Cox, A.J.; Kruglyak, S.; Saunders, C.T. Manta: Rapid detection of structural variants and indels for germline and cancer sequencing applications. *Bioinformatics* **2016**, *32*, 1220–1222. https://doi.org/10.1093/bioinformatics/btv710.
13. Cameron, D.L.; Schroder, J.; Penington, J.S.; Do, H.; Molania, R.; Dobrovic, A.; Speed, T.P.; Papenfuss, A.T. GRIDSS: Sensitive and specific genomic rearrangement detection using positional de Bruijn graph assembly. *Genome Res* **2017**, *27*, 2050–2060. https://doi.org/10.1101/gr.222109.117.
14. Jeffares, D.C.; Jolly, C.; Hoti, M.; Speed, D.; Shaw, L.; Rallis, C.; Balloux, F.; Dessimoz, C.; Bahler, J.; Sedlazeck, F.J. Transient structural variations have strong effects on

quantitative traits and reproductive isolation in fission yeast. *Nat Commun* **2017**, *8*, 14061. https://doi.org/10.1038/ncomms14061.
15. Chiang, C.; Layer, R.M.; Faust, G.G.; Lindberg, M.R.; Rose, D.B.; Garrison, E.P.; Marth, G.T.; Quinlan, A.R.; Hall, I.M. SpeedSeq: Ultra-fast personal genome analysis and interpretation. *Nat Methods* **2015**, *12*, 966–968. https://doi.org/10.1038/nmeth.3505.
16. Wong, P.; Wagner, J.A.; Berrien-Elliott, M.M.; Schappe, T.; Fehniger, T.A. Flow cytometry-based ex vivo murine NK cell cytotoxicity assay. *STAR Protoc* **2021**, *2*, 100262. https://doi.org/10.1016/j.xpro.2020.100262.
17. Rosenfeld, C.; Goutner, A.; Venuat, A.M.; Choquet, C.; Pico, J.L.; Dore, J.F.; Liabeuf, A.; Durandy, A.; Desgrange, C.; De The, G. An effect human leukaemic cell line: Reh. *Eur J Cancer (1965)* **1977**, *13*, 377–379. https://doi.org/10.1016/0014-2964(77)90085-8.
18. Eberhard, D.; Jimenez, G.; Heavey, B.; Busslinger, M. Transcriptional repression by Pax5 (BSAP) through interaction with corepressors of the Groucho family. *EMBO J* **2000**, *19*, 2292–2303, doi:cdd215.
19. Yamashita, M.; Kitano, S.; Aikawa, H.; Kuchiba, A.; Hayashi, M.; Yamamoto, N.; Tamura, K.; Hamada, A. A novel method for evaluating antibody-dependent cell-mediated cytotoxicity by flowcytometry using cryopreserved human peripheral blood mononuclear cells. *Sci Rep* **2016**, *6*, 19772. https://doi.org/10.1038/srep19772.
20. Casado-Garcia, A.; Isidro-Hernandez, M.; Oak, N.; Mayado, A.; Mann-Ran, C.; Raboso-Gallego, J.; Aleman-Arteaga, S.; Buhles, A.; Sterker, D.; Sanchez, E.G.; et al. Transient Inhibition of the JAK/STAT Pathway Prevents B-ALL Development in Genetically Predisposed Mice. *Cancer Res* **2022**, *82*, 1098–1109. https://doi.org/10.1158/0008-5472.CAN-21-3386.
21. Isidro-Hernandez, M.; Casado-Garcia, A.; Oak, N.; Aleman-Arteaga, S.; Ruiz-Corzo, B.; Martinez-Cano, J.; Mayado, A.; Sanchez, E.G.; Blanco, O.; Gaspar, M.L.; et al. Immune stress suppresses innate immune signaling in preleukemic precursor B-cells to provoke leukemia in predisposed mice. *Nat Commun* **2023**, *14*, 5159. https://doi.org/10.1038/s41467-023-40961-z.
22. Martin-Lorenzo, A.; Hauer, J.; Vicente-Duenas, C.; Auer, F.; Gonzalez-Herrero, I.; Garcia-Ramirez, I.; Ginzel, S.; Thiele, R.; Constantinescu, S.N.; Bartenhagen, C.; et al. Infection Exposure is a Causal Factor in B-cell Precursor Acute Lymphoblastic Leukemia as a Result of Pax5-Inherited Susceptibility. *Cancer Discov* **2015**, *5*, 1328–1343. https://doi.org/10.1158/2159-8290.CD-15-0892.
23. Rodriguez-Hernandez, G.; Hauer, J.; Martin-Lorenzo, A.; Schafer, D.; Bartenhagen, C.; Garcia-Ramirez, I.; Auer, F.; Gonzalez-Herrero, I.; Ruiz-Roca, L.; Gombert, M.; et al. Infection Exposure Promotes ETV6-RUNX1 Precursor B-cell Leukemia via Impaired H3K4 Demethylases. *Cancer Res* **2017**, *77*, 4365–4377. https://doi.org/10.1158/0008-5472.CAN-17-0701.
24. Rodriguez-Hernandez, G.; Opitz, F.V.; Delgado, P.; Walter, C.; Alvarez-Prado, A.F.; Gonzalez-Herrero, I.; Auer, F.; Fischer, U.; Janssen, S.; Bartenhagen, C.; et al. Infectious stimuli promote malignant B-cell acute lymphoblastic leukemia in the absence of AID. *Nat Commun* **2019**, *10*, 5563. https://doi.org/10.1038/s41467-019-13570-y.
25. Vicente-Duenas, C.; Janssen, S.; Oldenburg, M.; Auer, F.; Gonzalez-Herrero, I.; Casado-Garcia, A.; Isidro-Hernandez, M.; Raboso-Gallego, J.; Westhoff, P.; Pandyra, A.A.; et al. An intact gut microbiome protects genetically predisposed mice against leukemia. *Blood* **2020**, *136*, 2003–2017. https://doi.org/10.1182/blood.2019004381.

.

2 - Supplemental Tables:.

.

.

Table S1. Percentage of PD-1+ leukemic cells in the *Pax5*$^{+/-}$ and *Sca1-ETV6-RUNX1* mice with B-ALL.

.

Mouse genotype.	Mouse ID..	Leukemic cells in the bone marrow..	PD-1^{+} leukemic cells in the bone marrow..	Leukemic cells in the peripheral blood..	PD-1^{+} leukemic cells in the peripheral blood..
Pax5$^{+/-}$.	Q746.	94%.	0%.	80.61%.	0%..
	G977	91.49%	0,00%	70.87%	0,00%
	S931	94.1%	0,00%	91.09%	0,00%
	S906	94.99%	0,00%	90.20%	0,00%
	G065	ND	ND	69%	0,00%
	S678	96.2%	95.2%	13.82%	8.87%
	S864	96.93%	47.81%	89.77%	81.88%
	Q738	46.78%	44.68%	4.83%	4.80%
	G811	88.61%	72.17%	14.30%	14.20%
	G970	84.77%	81.59%	28.93%	26.05%
	S135	71.56%	27.15%	71.76%	23.81%
	G975	92.45%	73.07%	49.38%	26.33%
	S859	ND	ND	55.99%	48.26%
	S679	ND	ND	82.51%	74.64%
Sca1-EVT6-RUNX1	Q782	95.1%	0%	9.24%	0%
	M997	88.86%	85.9%	44.68%	40.61%
	Q783	45.16%	45.16%	14.20%	23.49%

ND: Not determined

Table S2. Childhood B-ALL xenograft cases.

Sample ID	*Tumor type*	*Tumor WGS and RNA-seq*	*PD-1 expression*	*PD-1+ cells (%)*
SJBALL019053_D1	ETV6::RUNX1	focal CNV: PAX5 del	-	0
SJBALL019053_R1	ETV6::RUNX1	focal CNV: PAX5 del	+	24
SJETV004796_D2	ETV6::RUNX1		-	0
SJETV010_D	ETV6::RUNX1	SJETV010_D (hg19)	+	32
SJALL057950_D1	PAX5alt	CNV: -9p with PAX5	+	5
SJALL056748_D1	PAX5alt	chr9.37002674.T.TCGGGCGAAGGAAGGGAGGGAAGGAAGGGAGGGAAGGAAGGGAGGGAAGGGAGGGAGGGAAGGGAGGGAGGGAAGGGAGGGAGGGAAGGGAGGGAGGGAAGGGAGGTATTCCCTC (PAX5 proteinIns: D193>GGNTSLPSLPSLPPFPPSLPSLPSLPSFPPFLPSLPSPD)	-	0
SJBALL015056_D1	PAX5 P80R	CNV: -9p with PAX5<br>chr9.37015168.G.C PAX5 P80R	+	17
SJBALL015077_D1	PAX5 P80R	chr9.37015166.CA.C PAX5 P80fs<br>chr9.37015168.G.C PAX5 P80R<br>They are biallelic variants	+	29
SJBALL059204_D1	Hyperdiploid		+	23
SJALL048483_D1	Hyperdiploid		+	100
SJBALL021950_D1	Hyperdiploid		+	7

Table S3. Clinical characteristics of B-ALL patients at diagnosis (upper table) or at relapse (lower table).

PD-1 EXPRESSION			
	POSITIVE	NEGATIVE	P value
**n**	11	17	
**SEX (MALE / FEMALE)**	7 / 4	8 / 9	0,260
**AGE**	10,8 ± 1,68	7,8 ± 0,99	0,1321
**ABSOLUTE BLAST COUNT (/µL)**	134,6 ± 75,0	34,9 ± 8,7	0,1514
**RISK GROUP**	1 HIGH / 10 INTERMEDIATE	1 HIGH / 16 INTERMEDIATE	0,664
**RELAPSE**	1 OUT OF 11	1 OUT OF 17	0,664

PD-1 EXPRESSION			
	POSITIVE	NEGATIVE	P value
**n**	7	12	
**SEX (MALE / FEMALE)**	6 / 1	2 / 10	0,006
**AGE**	10,6 ± 1,87	11,6 ± 1,33	0,8987
**TIME**	4 EARLY[1] / 3 LATE[2]	6 EARLY[1] / 6 LATE[2]	0,57
**LOCATION**	6 ISOLATED[3] / 1 COMBINED[4]	10 ISOLATED[3] / 2 COMBINED[4]	0,704

[1]More than 18 months after diagnoses and less than 6 months after end of therapy. [2]More than 6 months after end of therapy. [3]CNS or testes leukemia with no marrow involvement. [4]CNS or testes leukemia plus marrow involvement. Criteria from the Spanish Society of Hematology and Oncology- Acute Lymphoblastic Leukemia Relapse Clinical Guidelines.

* One-sided Fisher's exact test. ** Two-sample Wilcoxon rank-sum (Mann-Whitney) test.

Table S4. Fluorochrome-conjugated monoclonal antibodies used for flow cytometry-based evaluation of PD-1 expression on human B-ALL blasts

Monoclonal antibody reagents were combined in two antibody panels (A and B) each stained in parallel in a separate sample aliquot.
APC, allophycocyanine; BV, brilliant violet; PacB, Pacific Blue; PerCP, peridinin-chlorophyll-protein; Cy, cyanine; PE, phycoerythrin; AF, alexa fluor; FITC, fluorescein isothiocyanate.

Marker	*Fluorochrome*	*Antibody clone*	*Antibody identifier*	*Panel*
CD10	APC	HI10a	BD Biosciences Cat..Nº 332777	A
CD16	BV711	3G8	BD Biosciences Cat. Nº 563127	B
CD19	BV786	SJ25C1	BD Biosciences Cat. Nº 563325	A, B
CD20	PacB	2H7	BioLegend Cat. Nº 302320	A, B
CD34	PERCPCy5.5	8G12	BD Biosciences Cat. Nº 347222	A
	PE CF594	581	BD Biosciences Cat. Nº 562383	B
CD38	BV510	HIT2	BD Biosciences Cat. Nº 303540	B
CD45	PERCPCy5.5<br>AF700	HI30	BioLegend Cat Nº 304028<br>BD Biosciences Cat Nº 560566	A
CD56	PE	C5.9	Cytognos Cat. Nº CYT-56PE	B
CD66c	PE	KOR-SA3544	Beckman Coulter Cat. Nº IM2357U	A
PD-1 (CD279)	PECy7	EH12.1	BD Biosciences Cat. Nº 561272	A, B
PD-L1 (CD274)	FITC	MIH1	BD Biosciences Cat. Nº 558065	A, B
FSV 780 Viability Marker			BD Biosciences Cat. Nº 565388	A, B

3- Supplemental Figures:

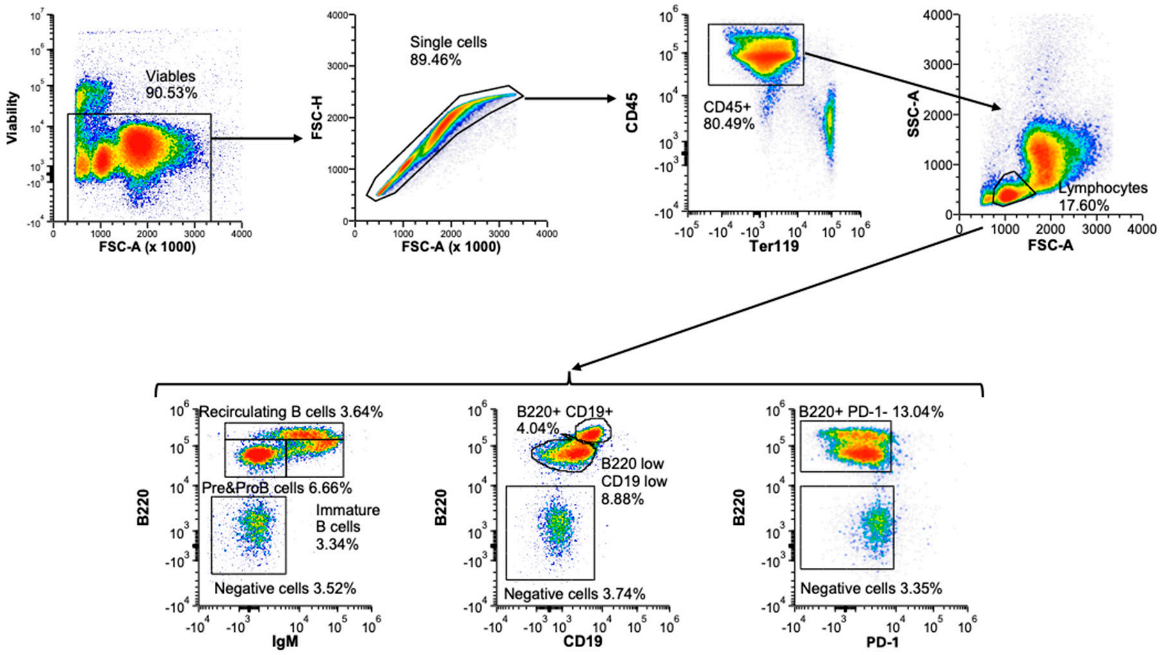

Figure S1. Gating strategy for cytometry analysis. Dead cells are excluded from the analysis by the Zombie NIR positive fluorescence; from this point on, only viable cells (90.53% in this case) are considered. Single cells (89.46%) were gated using forward scatter area (FSC-A) and height (FSC-H); this step allows us to exclude doblets and triplets in the following steps. CD45+ cells (80.49%) were selected to exclude erythroid cells (Ter119+), and lymphocytes (17.60%) were gated from CD45+ cells based on FSC-A and side scatter (SSC-A); FSC-A vs SSC-A allows us to identify lymphocytes and discriminates from myeloid populations. Further gating from lymphocytes was performed to identify specific B cell subpopulations, including recirculating B cells (3.64%), Pre/Pro B cells (6.66%), immature B cells (3.34%), B220+ CD19+ (4.04%), B220 low CD19 low (8.88%), and B220+ PD-1+ cells (13.04%). The percentage of each cell subpopulation relative to the total tissue cellularity is shown.

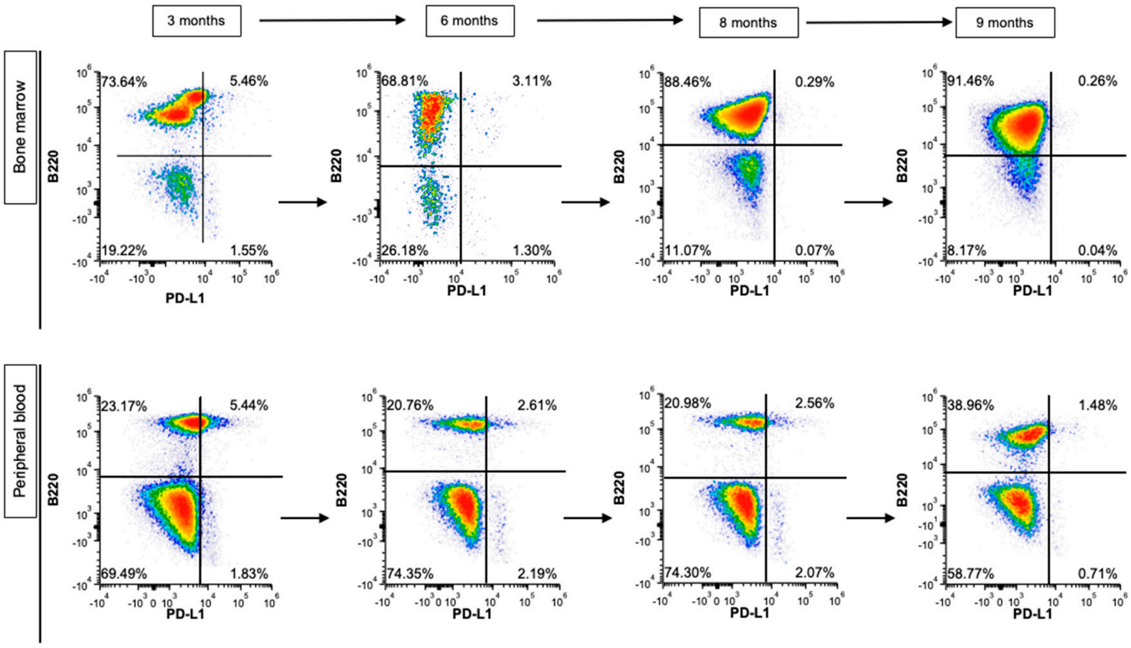

Figure S2. Spectral flow cytometry analysis of *Pax5*$^{+/-}$ B-ALL: B220 vs PD-L1 plots. No PD-L1 expression was detected by spectral flow cytometry.

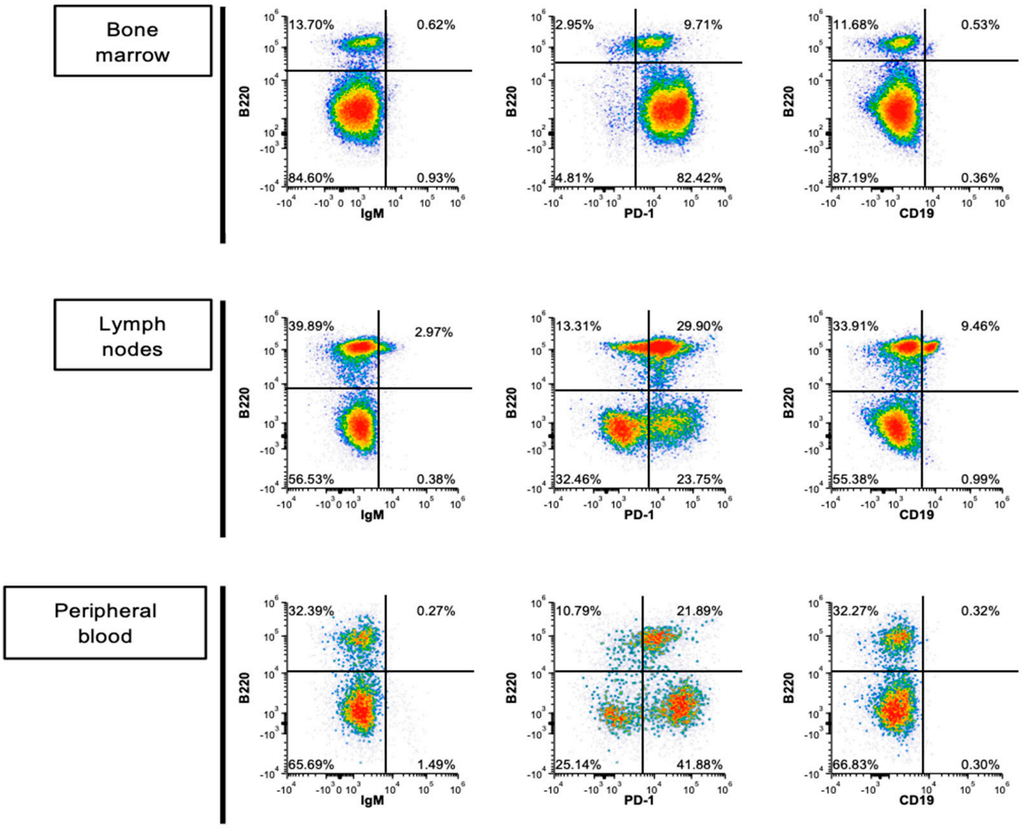

Figure S3A. Spectral flow cytometry analysis of *Pax5*$^{+/-}$ B-ALL.

A representative example of leukaemic cells (B220$^{+/-}$ PD1^{+} CD19^{-} IgM^{-}) in bone marrow, lymph nodes and peripheral blood of a diseased Pax5+/- mouse is shown.

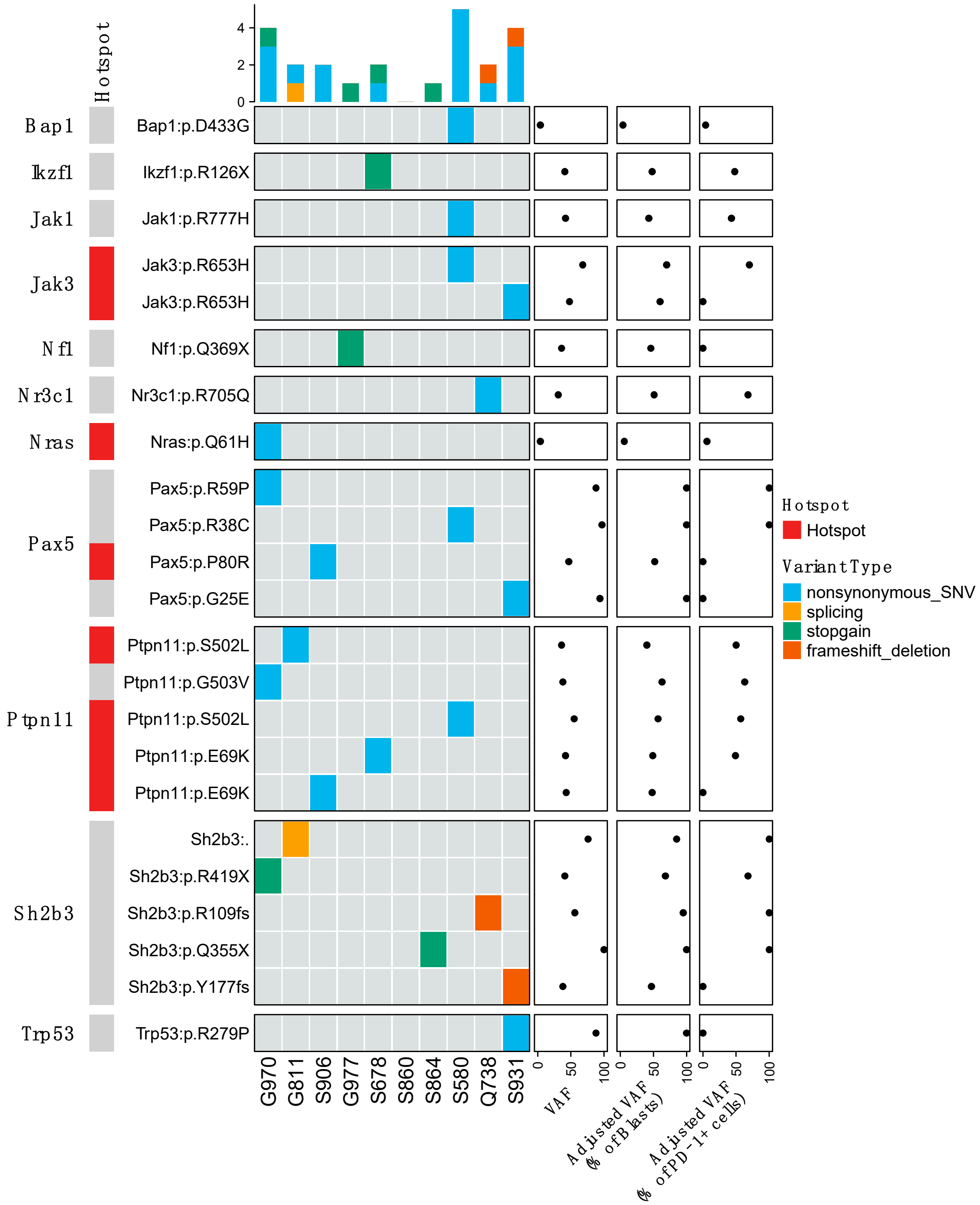

Figure S3B. Oncoprint of somatic single nucleotide mutations in a subset of leukemia associated genes across ten leukemia samples from *Pax5*$^{+/-}$ leukemic mice (G970, G811; S678; S864; S580; Q738 were PD-1+ and S906; G977; S860; S931 were PD-1-). Somatic mutations are clustered by gene. Previously known human or mouse leukemia hotspot mutations are highlighted (red). Tumor variant allele fraction (VAF) for each somatic single nucleotide mutation is shown in a dot plot on the right. Tumor variant allele fractions adjusted for fraction of leukemic blasts and fraction of PD-1^{+} leukemic cells are calculated by dividing un-adjusted VAF with either fraction of leukemia blasts or fraction of PD-1^{+} cells.

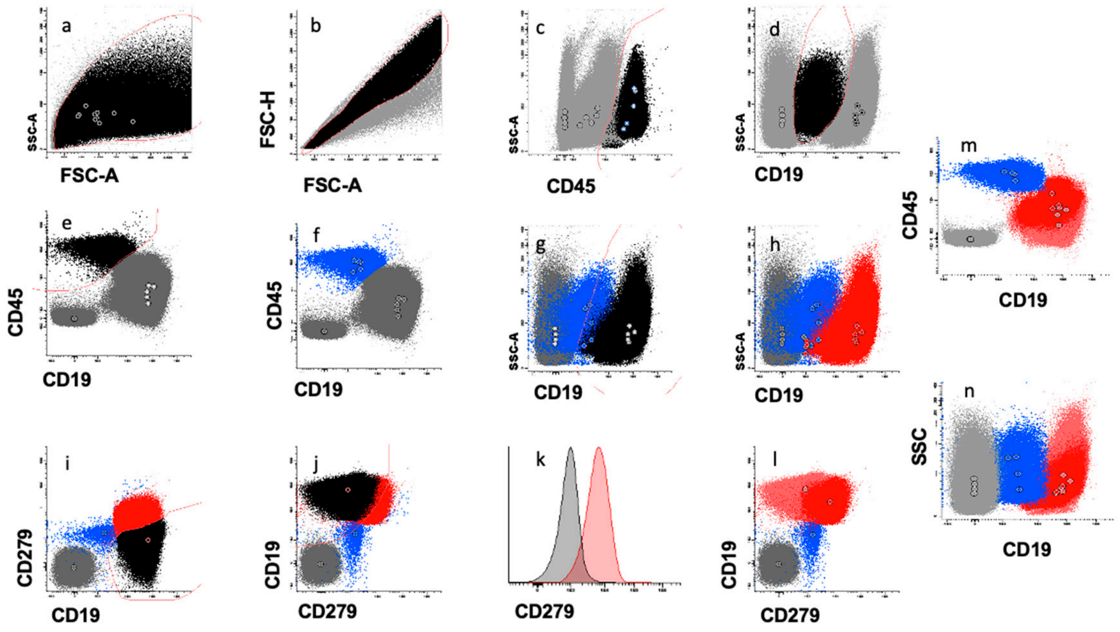

Figure S4. **Analysis strategy.** In panels a-b, the identification of nucleated cells (excluding debris and doublets) is shown. Within the nucleated cells, normal mouse cells are identified by $CD45^{++}$ and $CD19^{-/+dim}$ expression (panels c-f), and B-ALL cells are identified by their $CD45^{+dim}$ and $CD19^{++}$ expression profiles (panels g-h). Within B-ALL cells, $PD\text{-}1^{+}$ cells are identified in panels i-l. Mouse cells are depicted as blue dots, $PD\text{-}1^{-}$ and $PD\text{-}1^{+}$ B-ALL cells are depicted in light and dark red, respectively while negative controls cells (unstained sample) are represented by grey dots.

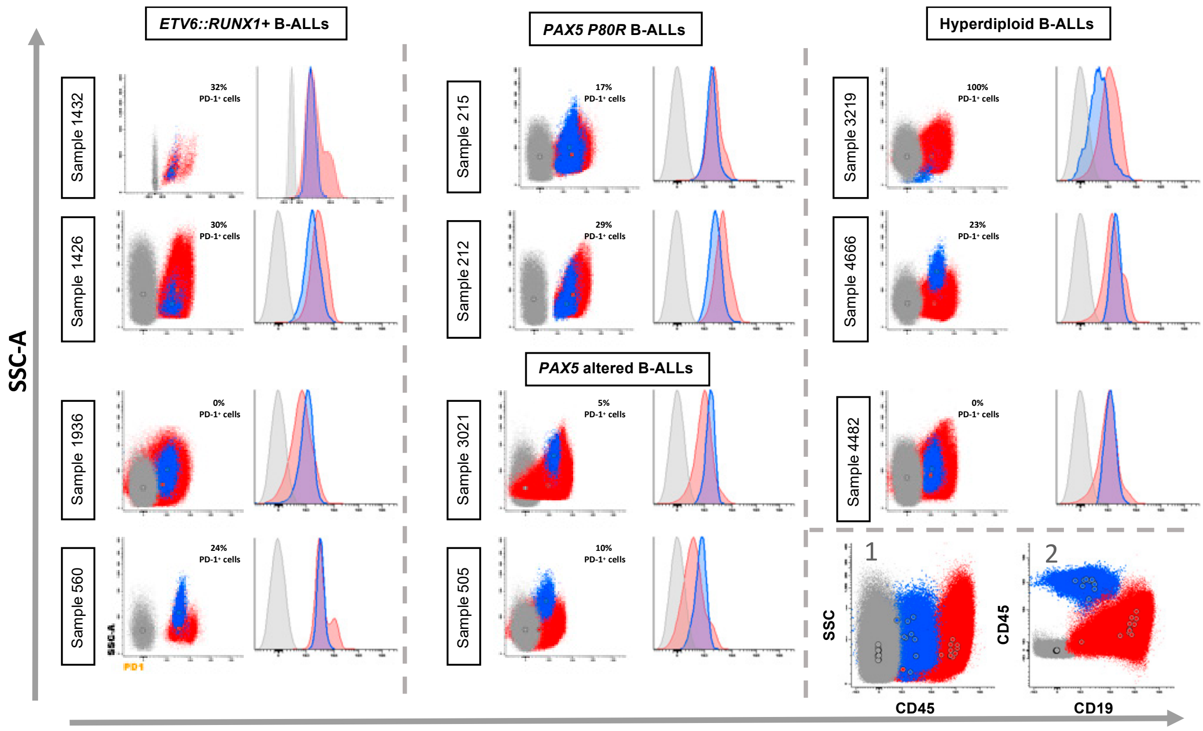

Figure S5. Spectral flow cytometry analysis of childhood leukemias growth in PDX models. Bivariate dot plots illustrating the expression of PD-1 in 11 samples from childhood leukemias growth in PDX models. Analysis by spectral flow cytometry of *Pax5-alt* B-ALL (n=4), *ETV6::RUNX1*+ B-ALL (n=4) and hyperdiploid B-ALL (n=3) confirmed the expression of PD-1 in the majority of B-ALL samples. Spleens of the recipient mice were used for the flow cytometric analysis and isolation of leukemic cells. Identification of B-ALL leukemic cells and internal negative and positive control cells was based on their uniquely distinct pattern of expression of the CD16, CD56, CD34, CD10, CD45, CD19, and CD20 markers. Leukemic PD-1+ B-ALL cells are depicted in red and mouse cells are depicted in blue while negative control cells (unstained sample) are represented by green dots.

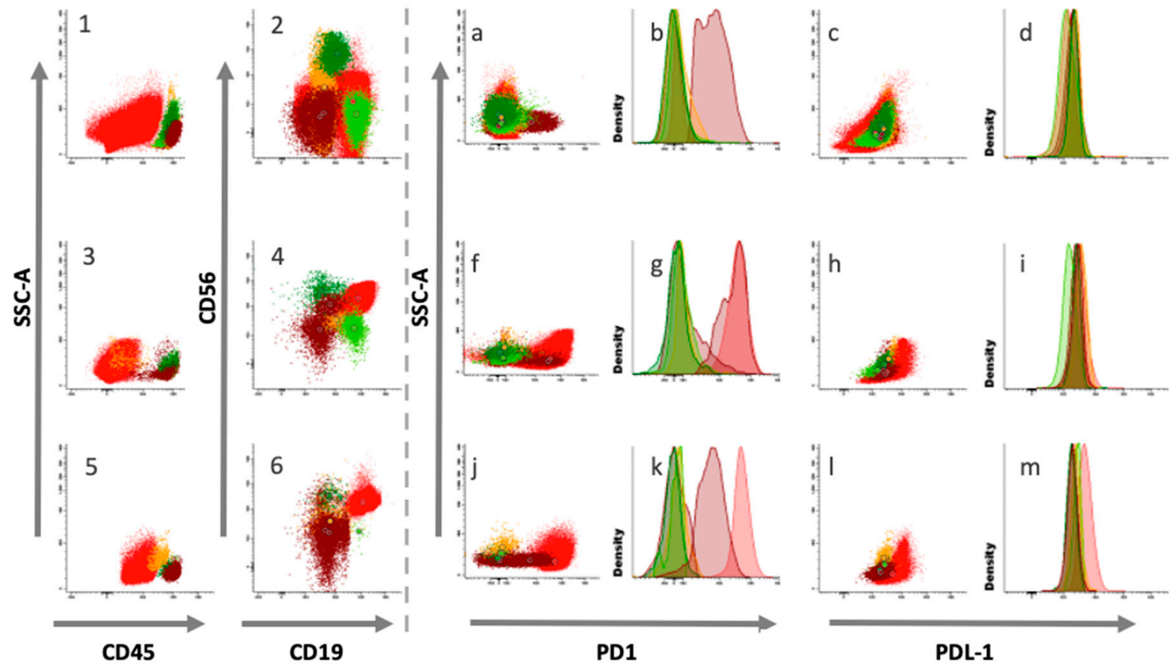

Figure S6. Bivariate dot plots and histograms illustrating the pattern of expression of PD-1 and PD-L1 in childhood B-ALL samples. Leukemic B-ALL cells are depicted in red, negative control cells including i.e., normal residual B cells and NK cells are depicted in dark and light green respectively, while positive control cells (i.e., a subset of normal residual T cells) are colored in brown. Other (PD1$^-$/PDL1$^-$) cell populations are represented in yellow orange.

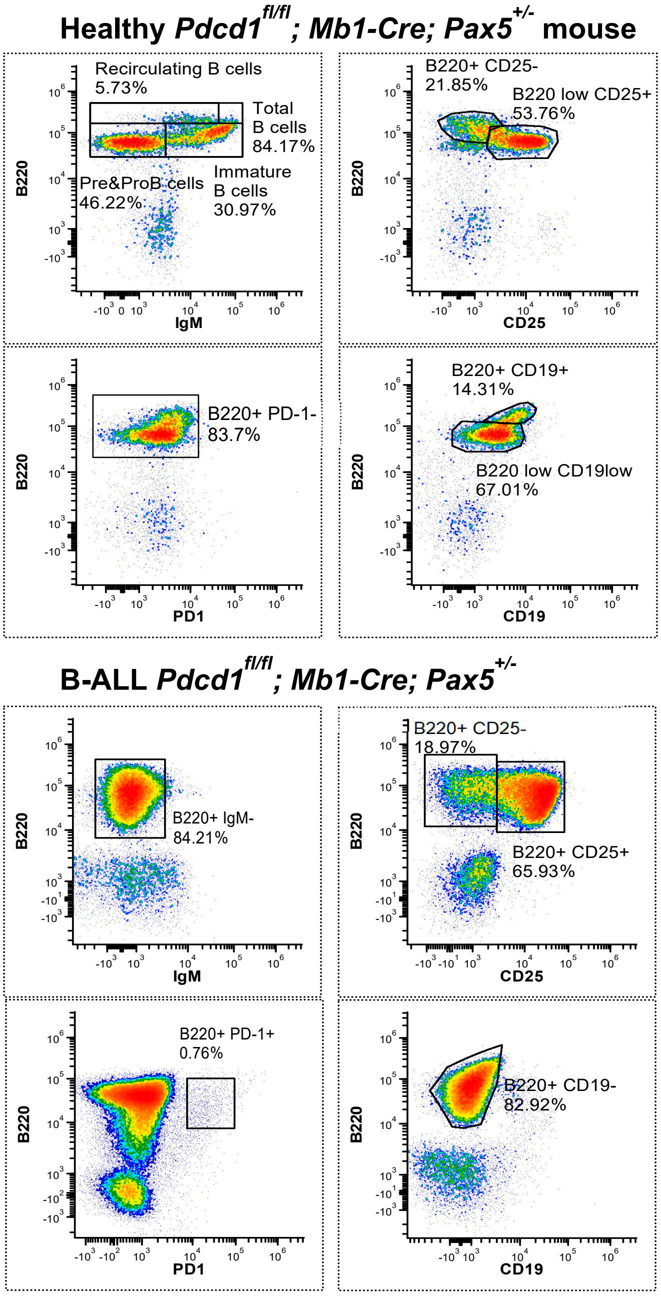

Figure S7A. PD-1 is not required for leukemogenesis in B-ALL. Representative spectral flow cytometry plots of the B cells subsets in the bone marrow of a healthy *Pdcd1*$^{fl/fl}$*;Mb1-Cre;Pax5*$^{+/-}$ mouse compared to same population in a B-ALL diseased *Pdcd1*$^{fl/fl}$*;Mb1-Cre;Pax5*$^{+/-}$ mouse. Neither leukemic nor healthy B-cells expressed PD-1.

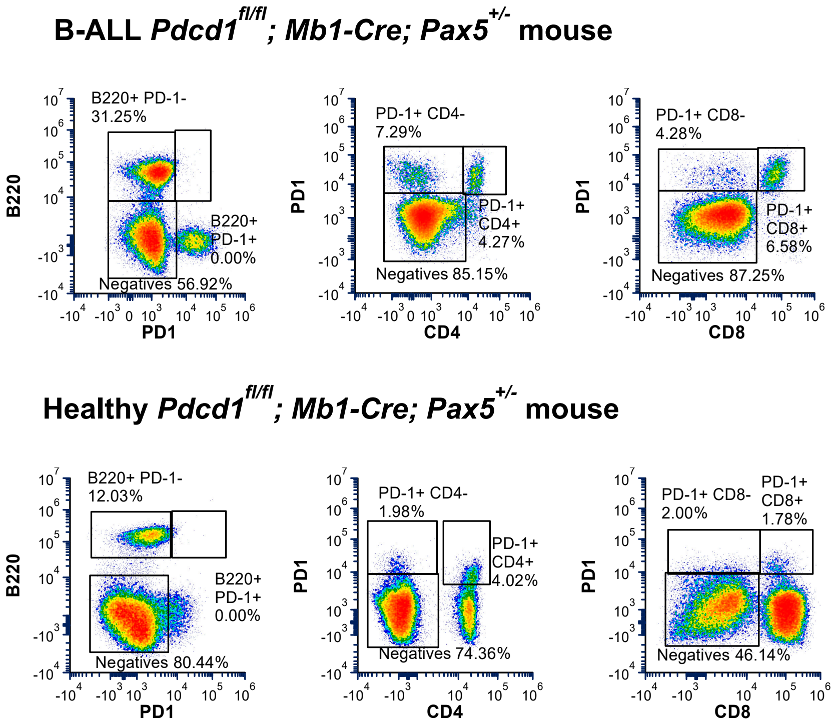

Figure S7B. Representative spectral flow cytometry plots of the expression of PD-1 in the B and T cells subsets in the bone marrow of a B-ALL diseased *Pdcd1*$^{fl/fl}$;*Mb1-Cre;Pax5*$^{+/-}$ mouse compared to same populations in a healthy *Pdcd1*$^{fl/fl}$;*Mb1-Cre;Pax5*$^{+/-}$ mouse. Neither leukemic nor healthy B-cells expressed PD-1, but the protein is expressed in T cell subsets (both CD8^{+} and CD4^{+} T cells).

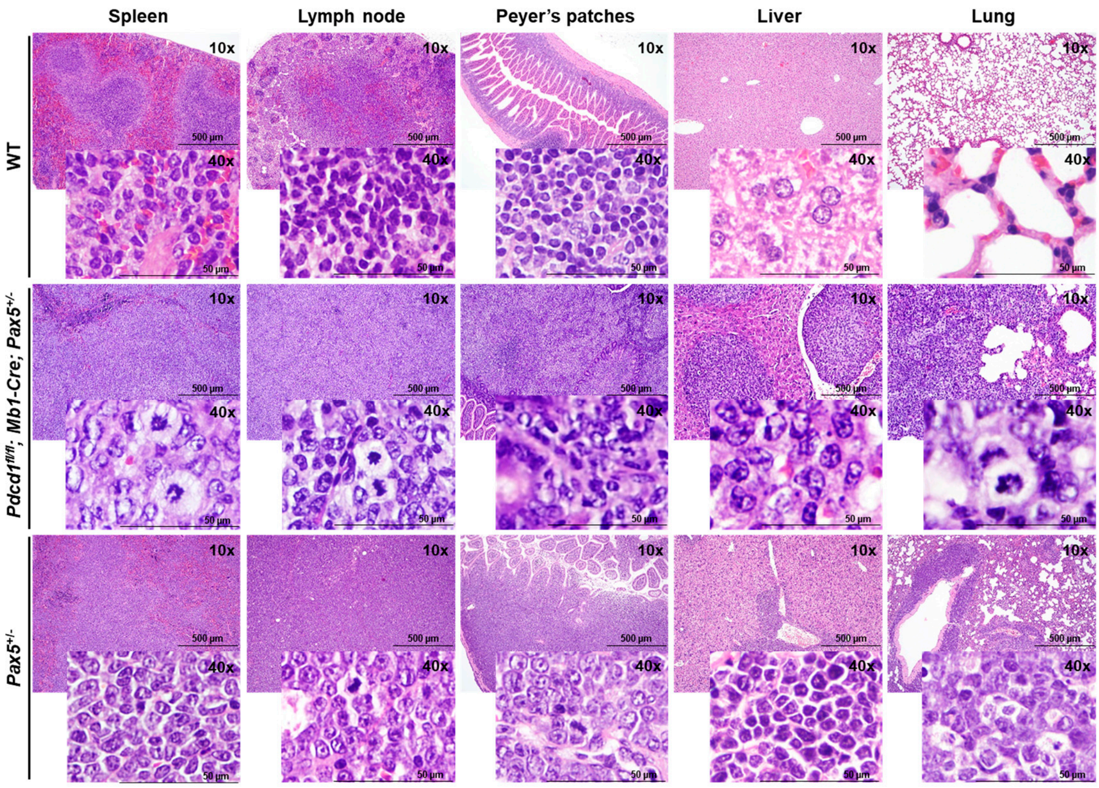

Figure S7C. Hematoxylin and eosin staining of *Pdcd1*$^{fl/fl}$*;Mb1-Cre;Pax5*$^{+/-}$ and *Pax5*$^{+/-}$ leukemic mice showed similar infiltration of blast cells in spleen, lymph nodes, Peyer's patches, liver and lung. Loss of normal architecture due to infiltrating leukemic cells can be seen. Tissues from a control littermate wild-type (WT) mouse are shown for reference. Magnification and corresponding scale bar are indicated.

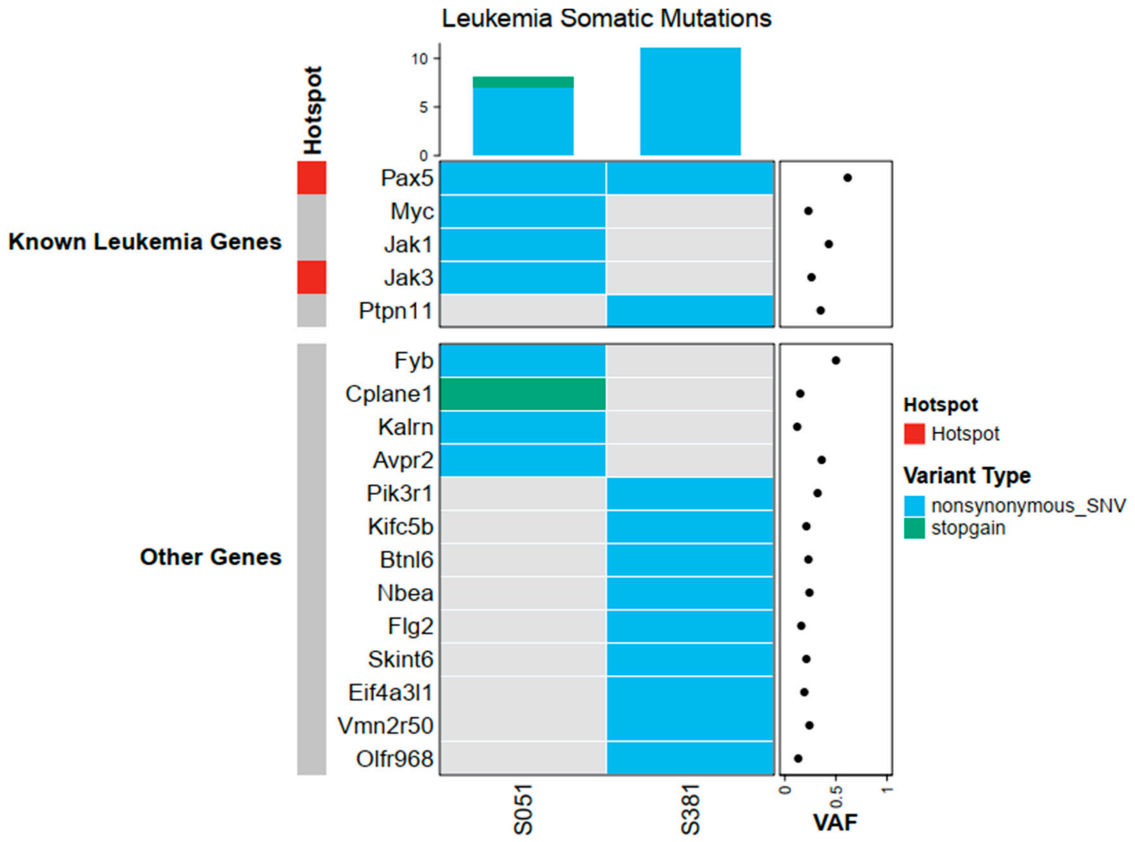

Figure S7D. Whole Genome Sequencing in leukemia in *Pdcd1*$^{fl/fl}$*;Mb1-Cre;Pax5*$^{+/-}$ mice. Oncoprint of somatic single nucleotide mutations and copy number alterations across 2 leukemia samples from *Pdcd1*$^{fl/fl}$*;Mb1-Cre;Pax5*$^{+/-}$ mice. Somatic alterations are clustered by gene. Tumor DNA was derived from whole leukemic BM, or lymph nodes (LN), while tail DNA of the respective mouse was used as reference germline material. The percentage of leukemic cells in the sequenced samples was 73% and 50%, respectively. Previously reported known human or mouse leukemia hotspot mutations are highlighted (red). Mean tumor variant allele fraction (VAF) for each single nucleotide mutation is shown on the dot plot on the right.

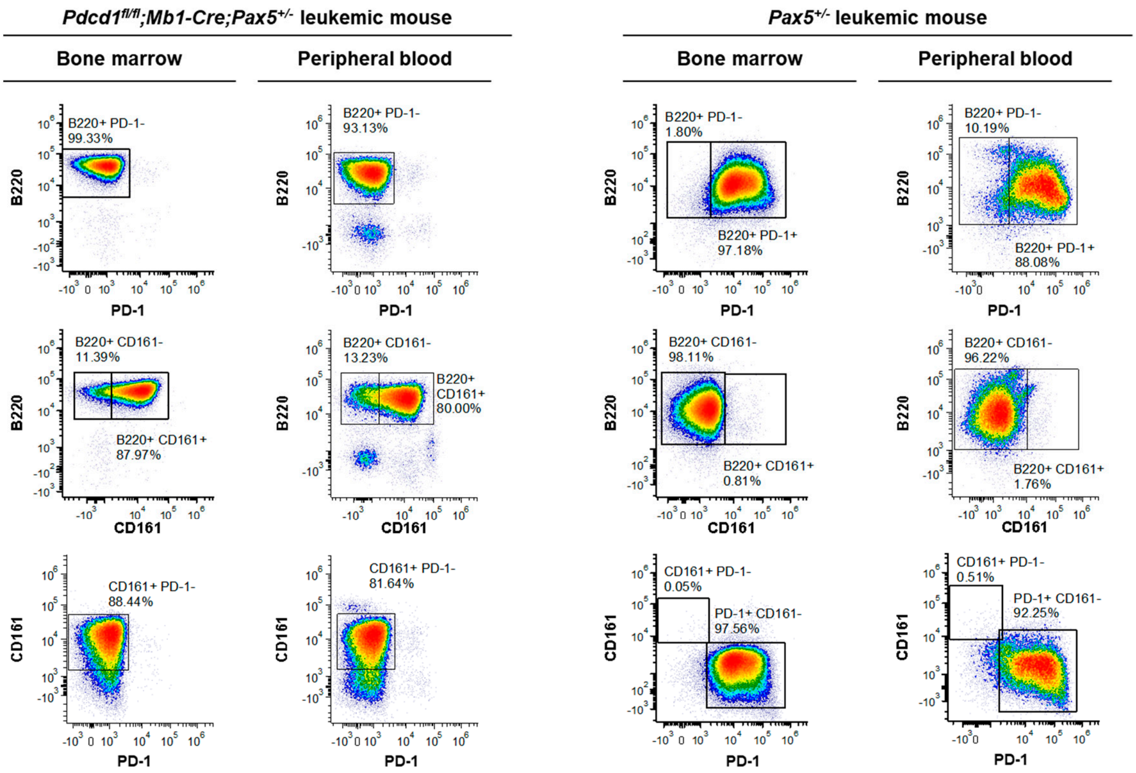

Figure S7E. Representative spectral flow cytometry plots of the leukemic population in the bone marrow (BM, first column) and peripheral blood (PB, second column) of a *Pdcd1fl/fl;Mb1-Cre;Pax5+/-* leukemic mouse expressing the CD161 marker compared to the leukemic population in a B-ALL diseased *Pax5+/-* mouse in BM (third column) and PB (fourth column), showing that in this case the leukemic cells express the PD-1 marker but not CD161.

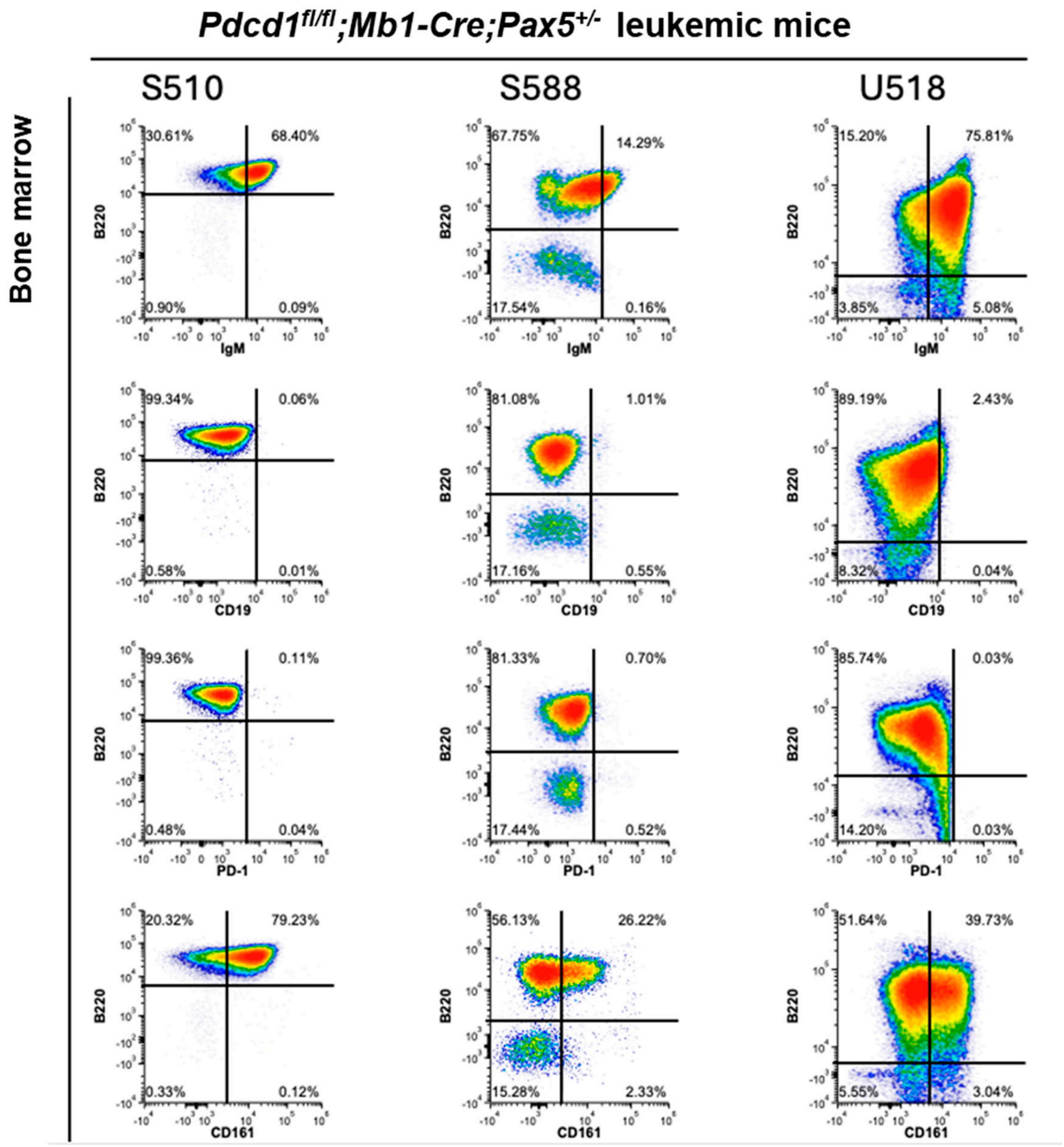

Figure S7F. B-ALL expressing CD161. Three cases of *Pdcd1fl/fl;Mb1-Cre;Pax5+/-* B-ALL mice (3 out of 13) expressed CD161, corroborating the notion that CD161 is only expressed when leukemic cells do not express PD-1 in order to evade immune surveillance. Spectral flow cytometry plots are shown.

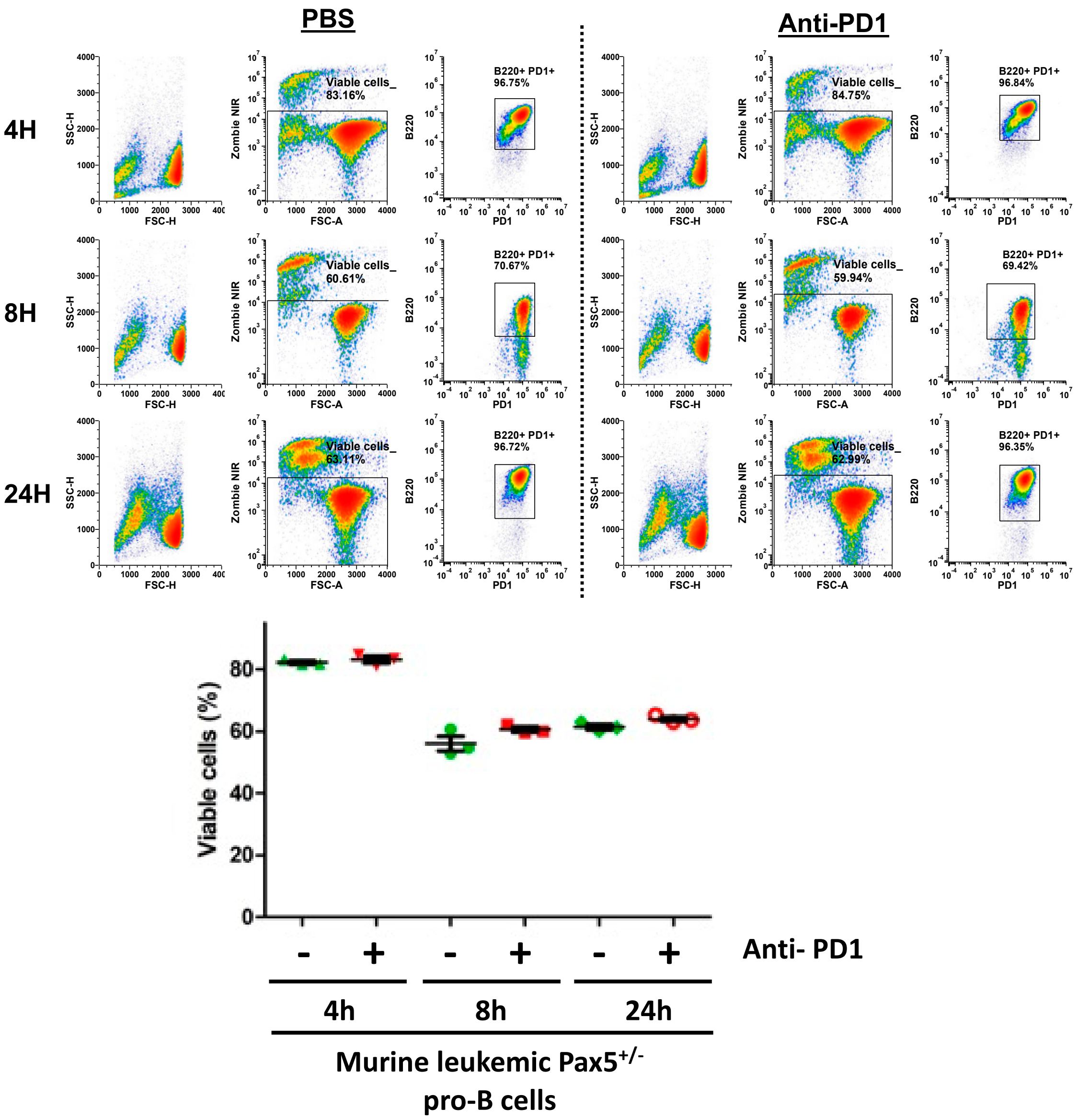

Figure S8. PD-1 targeting was not effective in an *in vitro* assay against leukemic cells. A. Murine leukemic *Pax5*$^{+/-}$ pro-B cells expressing PD-1 were cultured in the presence or absence of anti-PD1 for 4h (n=3), 8h (n=3), or 24 hours (n=3). The viability assay was performed on Cytek Northern Lights 2000 and data were analyzed with FCS Express software. No statistically significant differences, checked by unpaired t-test, were detected between the two conditions. Error bars represent the mean and standard deviation.

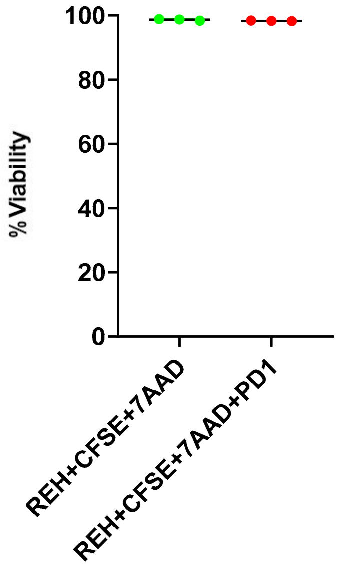

Figure S8B. Human *PAX5*-mutant B-cell leukemic REH cells (with the ETV6::RUNX1 translocation) were cultured in the presence (red) or absence (green) of an anti-PD1 IgG1 monoclonal antibody for 8 hours. Cell viability was determined by flow cytometry. Wilcoxon test p-value = 0.05.

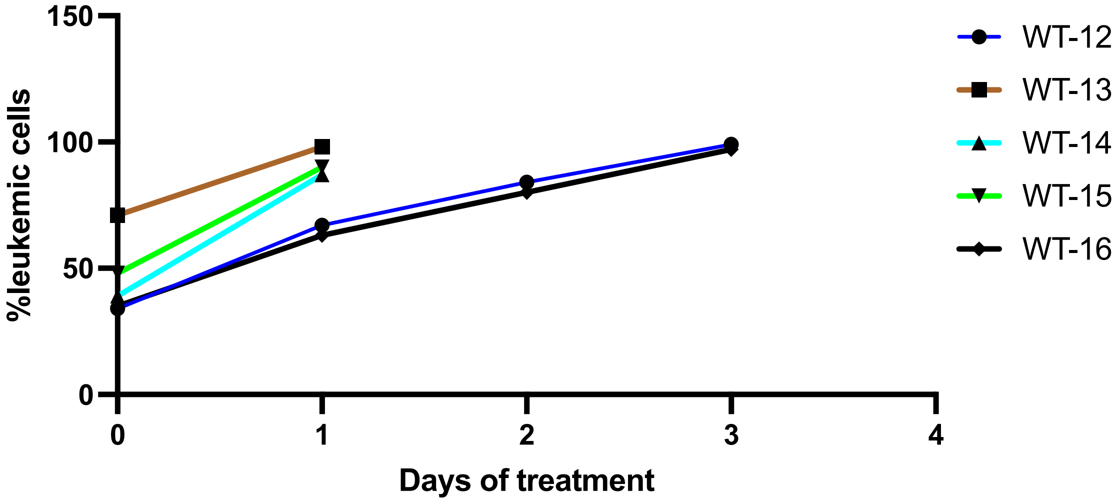

Figure S9. Leukemia evolution in PB of B-ALL-transplanted animals treated with placebo. **Figure S9A.** As a control to study the efficacy of the anti-PD1 treatment in B-ALL-injected WT mice, 5 of such B-ALL-injected animals were treated with injections containing only PBS. After confirmation of progression to leukemia by flow cytometry immunophenotyping of peripheral blood, placebo (PBS) treatment of the diseased animals was started. Repeated administration of PBS did not reduce leukemia burden in these B-ALL-transplanted animals. After 3 doses of PBS, all the animals had died due to leukemic cell accumulation.

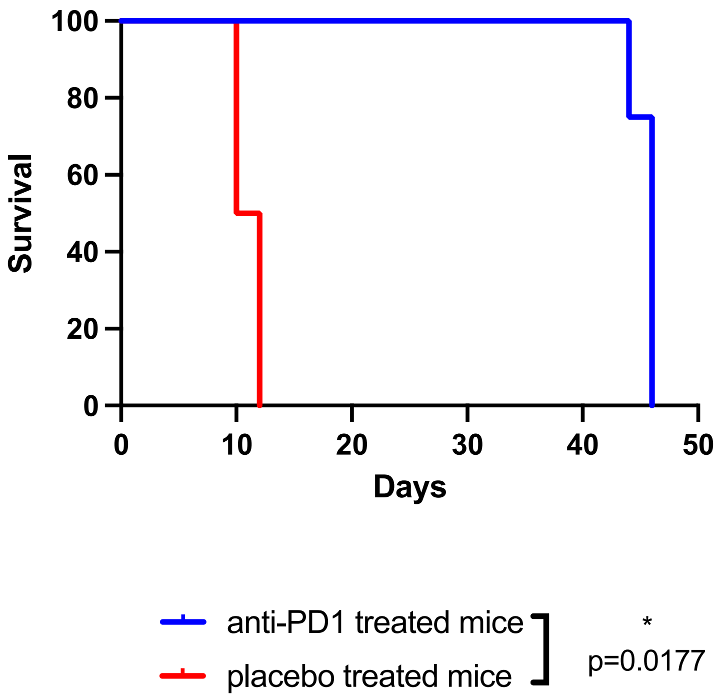

Figure S9B. B-ALL-specific survival curve of transplanted WT mice treated with anti-PD-1 therapy (blue line, n=6) and transplanted WT mice treated with placebo (red line, n=5). Log-rank (Mantel-Cox) p-value is shown.

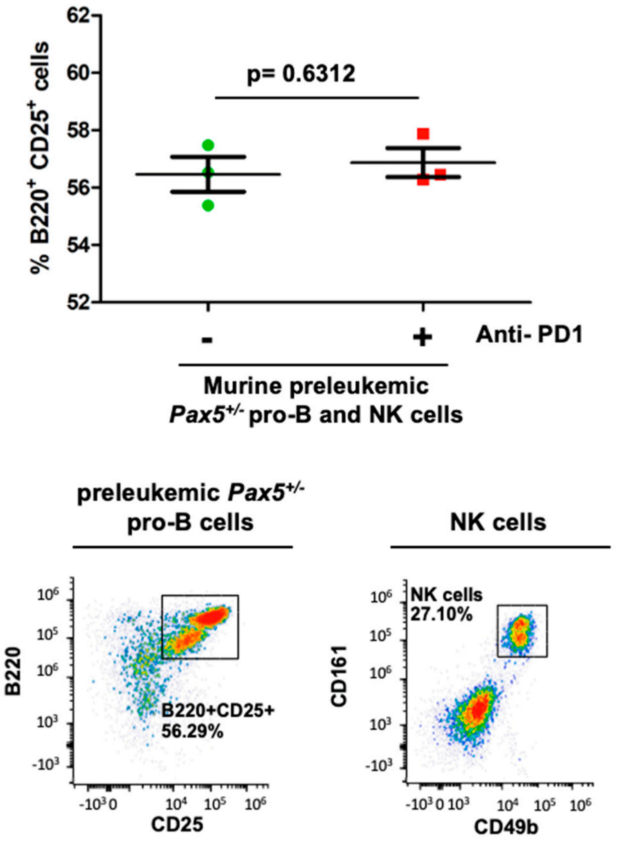

Figure S10. PD-1 targeting does not sensitize preleukemic *Pax5*$^{+/-}$ pro-B cells to NK cell-mediated killing. Murine preleukemic *Pax5*$^{+/-}$ pro-B cells (B220^{+} CD25^{+}) were co-cultured with murine NK cells (CD161^{+} CD49b^{+}) (1:1) in the presence of anti-PD1 antibody for 8h (n=3). The same cells cultured under the same conditions but with vehicle (PBS) were used as a control (n=3). An unpaired t-test was used to detect differences between the two culture conditions.
